# Supplementary figures and images for: Global gene expression and chromatin accessibility of the peripheral nervous system in animal models of persistent pain
Source: J Neuroinflammation. 2021 Aug 26;18:185. doi: 10.1186/s12974-021-02228-6 (PMC8390277; doi:10.1186/s12974-021-02228-6)

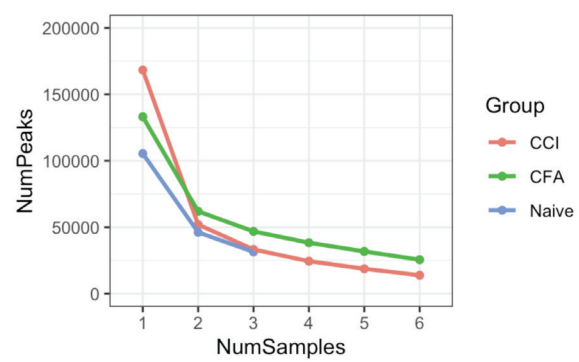

Supplement: Supplementary file 1 — Additional file 1: Supplemental Figure 1. Consistency of peaks among replicates. The graph shows the number of peaks identified in one or more samples. Due to the large number of accessible regions identified in only one sample, we included only regions that were identified in at least 50% of the samples within the study group. [file 12974_2021_2228_MOESM1_ESM.pdf]
